# Long non‐coding RNAs: Promising new targets in pulmonary fibrosis

**DOI:** 10.1002/jgm.3318

**Published:** 2021-02-11

**Authors:** Songzi Zhang, Hongbin Chen, Dayong Yue, Timothy S. Blackwell, Changjun Lv, Xiaodong Song

**Affiliations:** ^1^ Department of Cellular and Genetic Medicine, School of Pharmaceutical Sciences Binzhou Medical University Yantai China; ^2^ Department of Respiratory Medicine, Affiliated Hospital to Binzhou Medical University Binzhou Medical University Binzhou China; ^3^ Vanderbilt University Medical Center Nashville TN USA

**Keywords:** circular RNA, COVID‐19, lncRNA, microRNA, pulmonary fibrosis

## Abstract

Pulmonary fibrosis is characterized by progressive and irreversible scarring in the lungs with poor prognosis and treatment. It is caused by various factors, including environmental and occupational exposures, and some rheumatic immune diseases. Even the rapid global spread of the COVID‐19 pandemic can also cause pulmonary fibrosis with a high probability. Functions attributed to long non‐coding RNAs (lncRNAs) make them highly attractive diagnostic and therapeutic targets in fibroproliferative diseases. Therefore, an understanding of the specific mechanisms by which lncRNAs regulate pulmonary fibrotic pathogenesis is urgently needed to identify new possibilities for therapy. In this review, we focus on the molecular mechanisms and implications of lncRNAs targeted protein‐coding and non‐coding genes during pulmonary fibrogenesis, and systematically analyze the communication of lncRNAs with various types of RNAs, including microRNA, circular RNA and mRNA. Finally, we propose the potential approach of lncRNA‐based diagnosis and therapy for pulmonary fibrosis. We hope that understanding these interactions between protein‐coding and non‐coding genes will contribute to the development of lncRNA‐based clinical applications for pulmonary fibrosis.

## Graphical abstract

Different non‐coding RNAs (lncRNAs) exhibit different regulatory mechanisms because of their cellular localization, which, in turn, leads to their functional divergence in disease occurrence and progression. This review highlights how lncRNAs contribute to pulmonary fibrogenesis.

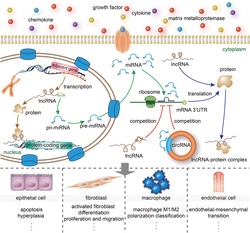

## 1 INTRODUCTION

Tissue fibrosis is a hallmark of chronic diseases in many organs, disturbing their architecture and resulting in dysfunction and failure.^1^, ^2^ Almost 45% of deaths are attributed to chronic fibroproliferative diseases, including pulmonary fibrosis.^3^ In addition to idiopathic pulmonary fibrosis (IPF) of unknown cause, progressive pulmonary fibrosis is the result of various factors, including environmental and occupational exposure, such as asbestos, mineral dust and radiation damage. Some rheumatic immune diseases, including systemic lupus erythematosus, rheumatoid arthritis, Sjogren's syndrome and dermatomyositis, can be accompanied by pulmonary fibrosis. Sudden public health events, such as the severe acute respiratory syndrome coronavirus, H5N1‐virus and the rapid global spread of the COVID‐19 pandemic, can also cause pulmonary fibrosis in many patients.^4^, ^5^ At present, the long‐term pulmonary consequences of COVID‐19 remain speculative and should not be assumed without appropriate prospective study. However, from the data of previous coronavirus infections such as severe acute respiratory syndrome and Middle East respiratory syndrome, it is suggested that there could be substantial fibrotic consequences following the COVID‐19 pandemic, and that even rare complications will have major health effects at the population level.^6^, ^7^

Despite significant progress in understanding disease pathogenesis, treatments for lung fibrosis are limited.^8^, ^9^ Two drugs, namely nintedanib and pirfenidone, are currently approved by the Food and Drug Administration for IPF treatment. Nintedanib is an intracellular tyrosine kinase inhibitor and pirfenidone is an anti‐fibrotic molecule that appears to work by reducing the proliferation and differentiation of fibroblasts. However, these drugs do not improve symptoms and are poorly tolerated.^10^, ^11^ Lung transplantation is the only treatment to improve life quality and the survival of pulmonary fibrosis patients.^12^ However, only a small proportion of patients can benefit from lung transplantation given that the demand outstrips the supply for organ transplants and immune rejection is still a serious challenge. As a result, a complete understanding of the molecular mechanisms implicated in pulmonary fibrosis pathogensis is urgently required to enable the development of new treatments.

Long non‐coding RNA (lncRNA) is defined as a regulatory non‐coding RNA of more than 200 nucleotides, including intergenic transcripts and enhancer RNA with sense or antisense orientation.^13^, ^14^ lncRNAs regulate numerous biological processes through different mechanisms in the nucleus and cytoplasm and are highly cell/tissue‐specific.^15^, ^16^ In this review, we focus on the molecular mechanisms and implications of lncRNAs during pulmonary fibrogenesis. Meanwhile, how lncRNAs cross‐talk with various types of RNAs, including microRNA (miRNA), circular RNA (circRNA) and mRNA, is also discussed. Finally, we propose the potential approach of lncRNA‐based diagnosis and therapy for pulmonary fibrosis.

## 2 LNCRNA DIRECTLY TARGETS PROTEIN‐CODING GENES TO REGULATE PULMONARY FIBROSIS

The act of transcription or the DNA element within the lncRNA locus are more likely to be the source of regulatory activity than the actual lncRNA itself.^17^, ^18^ Using a genome‐scale CRISPR–Cas9 activation screen of 10,000 lncRNA transcriptional start sites, Joung *et al*.^19^ reported that the majority of candidate loci appear to regulate nearby genes. In addition, lncRNA can form a RNA–protein complex with its binding protein to exert a positive or negative function.^20^ In pulmonary fibrosis, lncRNAs participate in the pathologic development of fibrosis by distinct mechanisms in different cell types, such as epithelial cells, fibroblasts and macrophages, etc.

### 2.1 lncRNA in epithelial cells

The initial phase of pulmonary fibrosis is usually considered to be a consequence of repetitive micro‐injuries to the alveolar epithelium, leading to changes in the cell microenvironment, fibroblast activation and deposition of matrix components.^21^, ^22^ Gokey *et al*.^23^ have identified 21 altered lncRNAs using single‐cell RNA sequencing and MEG3 was found to be the most increased lncRNA in IPF epithelial cells. lncMEG3 positively correlates with basal cell markers TP63, KRT5, KRT17, KRT14 and ITGB4, and negatively correlates with normal alveolar type II cell markers ABCA3, SFTPC and SFTPB. Further studies demonstrated that the lncMEG3 influences abnormal epithelial cell differentiation and increased epithelial cell migration by regulating numerous genes, including TP63, STAT3, KRT14, YAP1, AXL, TP53, EZH2 and transforming growth factor (TGF)β.^23^

However, the initial molecular mechanism and the close communication between lung epithelial cells and other type cells for fibrogenesis still remains unclear. Recently, Fukushima *et al*.^24^ demonstrated that alveolar epithelium cells apoptosis is sufficient to cause fibrosis by dysregulating the expression of nuclear exosome targeting complex component Rbm7‐lncNEAT1 axis, triggering the epithelium apoptosis in Rbm7‐deficient mice, bleomycin‐induced fibrosis mice, nonhematopoietic (CD45^−^) cells and RBM7^−/−^ HEK293 cells. Further mechanistic studies revealed that highly expressed Rbm7 induces the degradation of lncNEAT1 paraspeckles and the mislocalization of BRCA1 that disperses from lncNEAT1 paraspekles, leading to impaired DNA repair and triggering apoptosis. The dying alveolar epithelium releases chemokines that recruit atypical monocytes to drive lung fibrosis.^24^ On the whole, injured alveolar epithelium cells cause changes in not only TGFβ and chemokines, but also other microenvironment factors, such as hypoxia and reactive oxygen species (ROS). The role of lncRNA telomeric repeat‐containing RNA (TERRA) was also investigated in H_2_O_2_‐treated alveolar epithelial cells, bleomycin‐treated mice lung fibrosis and IPF patients.^25^ Functionally, lncTERRA accelerates the fibrotic process by promoting alveolar epithelium apoptosis. Mechanistically, lncTERRA forms a stable complex with the telomere repeat factor TRF2 and telomere DNA repeats to accelerate IPF pathogenesis through telomeric and mitochondrial pathways. During lung fibrogenesis, lncTERRA prevents telomere elongation by inhibiting telomerase activity and reverse transcriptase, and this condition inhibits cell cycle progression. Proliferating cell nuclear antigen, cyclin D1 and cyclin E are the regulatory factors of telomerase activity. RNA interference on lncTERRA enhances the expression of these factors, and this improvement promotes cell growth. The mitochondrion is also identified as the lncTERRA regulator of the IPF process. RNA interference on lncTERRA can ameliorate the functions of mitochondria by improving mitochondrial morphology, the electron transport chain and mitochondrial membrane potential. The findings demonstrate that lncTERRA contributes to fibrotic process by causing telomere attrition and mitochondrial dysfunction, in which mitochondria‐associated genes (Bcl‐2 family, cytochrome *c*, caspase‐9 and caspase‐3), oxidative stress‐associated genes or products (ROS, superoxide dismutase and catalase) and senescence regulatory genes (P53 and p53‐upregulated modulator of apoptosis) are all involved and play different important roles.^25^ Targets and therapeutic interventions with respect to the dysfunction of organelles have been developed. Nandrolone decanoate‐targeted abnormal telomere shortening is currently in a clinical trial and targets several diseases, including IPF (NCT02055456).^2^ MitoQ, which is a mitochondrially targeted antioxidant, is currently used in middle‐aged and older adults (NCT02597023).^26^ lncRNAs provide new strategies for intervening in the functioning of these organelles.

lncAP003419.16 is highly expressed in TGFβ1‐treated alveolar epithelial cells and IPF patients,^27^ in which lncAP003419.16 facilitates pulmonary fibrosis via the mammalian target of rapamycin (mTOR) signaling pathway dependent on its adjacent gene ribosomal protein S6 kinase B‐2 (RPS6KB2). Given that the mTOR signaling pathway and RPS6KB2 are involved in the process of aging and IPF,^28^, ^29^ it was proposed that lncAP003419.16 may predict an increased risk of aging‐associated IPF. Ageing is a crucial risk factor for pulmonary fibrosis independent of genetics and environment because most patients are older than 60 years.^30^, ^31^ Numerous lncRNAs influence the molecular processes that underlie age‐associated phenotypes.^32^, ^33^ However, additional credible evidence is needed to confirm that lncAP003419.16 predicts the risk of IPF because of the limited number of 20 patients investigated so far. In addition, the A549 cell model is easily questioned. A549 is often regarded as a normal representative of type II human alveolar epithelial cells,^34^, ^35^ although it is still a human lung adenocarcinoma cancer cell line. The characterization of fibrosis and cancer, such as the metabolism process and specific markers, is different.

Other lncRNAs in epithelial cells are lncRNAs‐uc.77 and 2700086A05Rik, which directly target zinc finger E‐box‐binding homeobox 2 and homeobox protein Hox‐A3 to promote paraquat‐induced pulmonary fibrosis by enhancing epithelial–mesenchymal transition (EMT) with increased α‐smooth muscle actin (SMA) and vimentin and reduced E‐cadherin.^36^ Overall, because the current strategy for treating fibrosis is mainly focused on fibroblasts, these lncRNAs, as initial mediators in fibrotic pathogenesis, provide promising new targets in epithelial cells for the early treatment of fibrosis.

### 2.2 lncRNA in fibroblasts

The fundamental pathogenic hallmarks of pulmonary fibrosis are uncontrolled proliferation and the high migration of activated fibroblasts. In pulmonary fibrosis, Song *et al*.^37^ and Cao *et al*.^38^ first identified 22 differentially expressed lncRNAs in a rat model of bleomycin‐induced lung fibrosis. Here, it should be noted that differentially expressed lncRNAs are very different in different platforms. There are many reasons for this, including the detection method (RNA‐sequencing or microarray), sample selection (cell type, rat, mice, patient blood or tissue) and the development of detection technology, and so on. These lncRNAs are roughly classified as sense‐exon‐overlap, sense‐intron‐overlap, antisense‐exon‐overlap, antisense‐intron‐overlap, bidirectional and intergenic according to their positions in genes. Among these lncRNAs, 15 lncRNAs show 90% sequence similarity to exons of protein‐coding genes spread across different chromosomes.^37^ The working mechanism of lncITPF is further elucidated in fibroblasts from IPF patients and human embryonic lung fibroblasts.^39^ lncITPF is transcribed from the tenth intron to the eleventh exon of its host gene integrin b‐like 1 (ITGBL1) and upregulated in the nucleus, which indicates that lncITPF regulates ITGBL1 transcription. ITGBL1 contains 10 repeats of epidermal growth factor‐like domain and encodes a β integrin‐related protein TIED; its high expression facilitates metastasis and invasion and correlates with poor survival in cancer patients.^40^ In pulmonary fibrosis, high ITGBL1 expression increases the fibrotic markers, such as a‐SMA, vimentin and collagen, and promotes myofibroblast proliferation and migration, thus resulting in fibrogenesis. The pro‐fibrotic role of lncITPF depends on ITGBL1 by enhancing H3 and H4 histone acetylation of the ITGBL1 promoter, in which lncITPF forms an RNA–protein complex with its direct binding protein heterogeneous nuclear ribonucleoprotein L (hnRNP L) to promote the activity of ITGBL1 promoter. However, lncITPF and ITGBL1 do not share the same promoter and are not co‐transcribed. In addition, numerous factors can lead to a change of lncRNA expression, such as chromosome opening, DNA damage and transcription factors. For lncITPF, the transcription factors smad2/3 bind over the lncITPF promoter and activate lncITPF transcription. Meanwhile, TGFβ1 also can activate lncITPF promoter activity. This condition indicates that the pro‐fibrotic function of lncITPF is mediated by the upstream TGFβ1‐smad2/3 signaling pathway.^39^ Except for the transcription factors smad2/3, other highly expressed transcription factors, such as E2F1 and YBX1, also can upregulate lncMALAT1 expression in the peripheral blood of IPF patients.^41^

Similarly, lncRNA NONMMUT028949.2 (lnc949) is also transcribed from a sequence within its host gene FK506 binding protein 5 (FKBP5) and exerts its pro‐fibrotic function by inhibiting FKBP5 expression. Unlike lncITPF, lnc949 is located in the cytoplasm and regulates FKBP5 post‐transcriptionally^42^; this difference illustrates the different regulatory mechanisms involving lncRNAs in pulmonary fibrosis. Both of these lncRNAs can promote pulmonary fibrosis by enhancing fibroblast proliferation and migration. RNA interference on lncITPF or lnc949 all can block fibrogenesis both *in vivo* and *in vitro*, which indicates their potential as therapeutic targets for IPF.

Certain studies have confirmed an incidence of lung cancer of 4.8%–48% and, without pulmonary fibrosis, of 2.0%–6.4%^43^; thus, IPF has been considered a precancerous condition or another form of cancer.^44^, ^45^ p53 is an important tumor gene because approximately 50% mutated p53 gene appear in tumors.^46^ Recently, lncRNA cyclin‐dependent kinase inhibitor‐2B‐antisense RNA 1 (CDKN2B‐AS1) in IPF patients was reported to predict lung cancer by regulating the p53‐signaling pathway. lncCDKN2B‐AS1 is reduced in the peripheral blood of IPF patients, and its neighboring gene CDKN2A is decreased simultaneously.^47^ CDKN2A, which is an important anti‐oncogene, encodes the p16INK4a and p14ARF proteins involved in the regulation of cell proliferation, survival and senescence.^48^, ^49^ This gene also contains an alternate open reading frame with a transcript that acts as a stabilizer of p53.^50^ lncCDKN2B‐AS1 controls the transcription of CDKN2A and thus regulates the biological functions p53. Accordingly, blocking abnormal cell proliferation and migration is an important treatment strategy in either cancer or IPF.

### 2.3 lncRNA in macrophages

Inflammatory cells are considered to contribute to the fibrotic pathogenesis by modulating the epithelial–fibroblast microenvironment, in which macrophages are one of the main effector cells and play roles in inflammation and fibrosis.^51^, ^52^ The alternative macrophage activation (M2) marker CD163 is observed in lung macrophages from IPF patients and Ly6Chi monocytes that can promote the pulmonary fibrosis progression.^53^ One lncRNA, namely Malat1, is reported to control pulmonary fibrosis in association with macrophage activation. Its expression is distinctly altered in differentially activated macrophages, in which Malat1 knockdown represses lipopolysacchride (LPS)‐induced M1 macrophage activation, whereas Malat1 knockdown elevates IL‐4‐activated M2 differentiation.^54^ Nuclear factor kappa B (NF‐κB) subunit p65 can bind to the Malat1 promoter. Thus, Malat1, which is a direct transcriptional target of LPS‐induced NF‐κB activation, increases pro‐inflammatory cytokines TNF‐α, IL‐6 and IL‐12 depending on target gene Clec16a (associated with aberrant inflammation gene) in LPS‐treated macrophages. By contrast, downregulated Malat1 promotes IL‐4‐activated pro‐fibrotic M2 differentiation with increased expression of M2 phenotypic markers arginase 1 (Arg‐1) and YM‐1, accompanied by the induction of mitochondrial pyruvate carriers, oxidative phosphorylation and an increased inmannose receptor C‐type 1 and oxygen consumption rate. Overall, Malat1 causes a change in the microenvironment by triggering macrophage activation to control lung fibrosis.^54^

Silicosis is a progressive pulmonary fibrosis and is initiated via the phagocytosis of silica particles by alveolar macrophages. An alteration profile of lncRNAs has been investigated in lung tissues of silica‐induced rats.^55^ Upregulated lncRNA LOC103691771 in the silicotic rat lung was further confirmed to activate macrophages and promote fibroblast differentiation via regulation of the TGFβ1‐Smad2/3 signaling pathway.^56^ Thus, investigations of lncRNA in macrophages will modulate the alveolar microenvironment, which will ameliorate pulmonary fibrosis.

### 2.4 lncRNA in endothelial cells

Lineage tracing experiments have identified endothelial cells as one of the sources of fibroblasts/myofibroblasts, termed endothelial‐mesenchymal transition (EndMT), which contributes to fibrotic distortion in IPF. EndMT can trigger the process of pulmonary fibrosis after PM_2.5_ exposure. Recently, a lncRNA microarray analysis revealed that 201 lncRNAs are upregulated and 108 lncRNAs are downregulated in the lung tissue of Balb/c mice exposed to PM_2.5_. The mechanism of EndMT and the upregulated lncRNA Gm16410 was further investigated and it was found that lncRNA Gm16410 mediates PM2.5‐induced EndMT by regulating the TGF‐β1/Smad3/p‐Smad3 pathway, which highlights the potential of lncRNAs to promote pulmonary fibrosis under environmental pollution.^57^

The above‐mentioned lncRNAs, located in various cells that directly target protein‐coding genes to regulate pulmonary fibrosis, are listed in Table 1.

**TABLE 1:** lncRNAs regulate pulmonary fibrosis through protein‐coding genes

lncRNA	Regulation	Model	Target gene	Signal pathway	Cell type	Reference
MEG3	Up	IPF patients donor lung	TP63, STAT3, KRT14, YAP1, AXL, TP53, EZH2 and TGFβ		Epithelial cell	Gokey *et al*.^23^
NEAT1	Down	Rbm7‐deficient mouse, bleomycin‐induced fibrosis mouse, nonhematopoietic (CD45‐) cells and RBM7−/− HEK293 cells	Rbm7, BRCA1		Epithelial cell	Fukushima *et al*.^24^
AP003419.16	Up	Blood samples from IPF patients and TGF‐β1‐treated type II alveolar epithelial cell A549 cell	RPS6KB2	mTOR	Epithelial cell	Hao *et al*.^27^
Uc.77	Up	Paraquat‐induced fibrosis mouse, primary human bronchial epithelial cell and human lung epithelial A549cell	Zeb2	EMT	Epithelial cell	Sun *et al*.^36^
05Rik	Up	Paraquat‐induced fibrosis mouse, primary human bronchial epithelial cell and human lung epithelial A549cell	Hoxa3	EMT	Epithelial cell	Sun *et al*.^36^
AJ005396	Up	Bleomycin‐induced fibrosis rat	Collagen α 1 type XI			Cao *et al*.^38^
S69206	Up	Bleomycin‐induced fibrosis rat	Rat mast cell protease 1 precursor (RMCP‐1)			Cao *et al*.^38^
ITPF	Up	Bleomycin‐ induced fibrosis rat, TGF‐β‐treated fibroblast MRC‐5 and blood samples from IPF patients	ITGBL1	TGF‐β‐Smad2/3 ‐hnRNP L	Fibroblast	Song *et al*.^39^
NONMMUT028949.2 (lnc949)	Up	Bleomycin‐induced fibrosis mouse and mouse lung fibroblast cell line L929	FKBP5		Fibroblast	Li *et al*.^42^
CDKN2B‐AS1	Down	IPF patients	CDKN2A	P53	Fibroblast	Du *et al*.^47^
Malat1	Up	LPS treated macrophage	Clec16a	Pro‐inflammatory response	Macrophage	Cui *et al*.^54^
Malat1	Down	IL‐4 treated macrophage	Hexokinases	Glucose metabolism	Macrophage	Cui *et al*.^54^
LOC103691771	Up	Silica‐induced rat TGF‐β1‐induced fibroblast,	TGFβ1	TGFβ1‐Smad2/3	Fibroblast	Cai *et al*.^56^
Gm16410	Down	Balb/c mouse and mouse pulmonary vascular endothelial cells by PM_2.5_ exposure		TGF‐β1/Smad3	Vascular endothelial cell	Ma *et al*.^57^

## 3 LNCRNA DIRECTLY TARGETS NON‐CODING GENES TO REGULATE PULMONARY FIBROSIS

High‐throughput technologies reveal that less than 2% of the human genome encodes protein, with the majority of the genome comprising ncRNAs that are not translated into proteins.^58^ Among these ncRNAs, regulatory ncRNAs including miRNA, lncRNA and circRNA can function as vital regulators of gene expression. The abnormally expressed miRNAs account for approximately 10% of total RNAs in the lungs of IPF patients.^59^, ^60^ CircRNA is the only circular RNA in the ncRNA family and is formed through head‐to‐tail splicing of mRNA or lncRNA exons.^61^ Bachmayr‐Heyda *et al*.^62^ analyzed circular and linear RNA expression and proposed that the ratio of circular to linear RNA isoforms is always lower in tumors and lung fibrosis compared to non‐diseased samples. Their cross‐talk regulates the pulmonary fibrosis pathogenesis.

### 3.1 lncRNA functions as a reservoir for miRNA

Emerging studies have shown that lncRNA can function as a reservoir for miRNA to regulate lung fibrogenesis. lncDNM3OS is identified as a reservoir for miR‐199a‐5p, miR‐199a‐3p and miR‐214‐3p in pulmonary fibrosis.^63^, ^64^ lncDNM3OS is a fibroblast‐specific downstream effector of TGFβ signaling that is transcribed from the negative strand of its host gene dynamin‐3 gene. lncDNM3OS promotes pulmonary fibrosis in *trans* by producing three miRNAs, namely miR‐199a‐5p, miR‐199a‐3p and miR‐214‐3p, which affect the major components Smad and non‐Smad in the TGFβ signal pathway. Among these miRNAs, miR‐199a‐5p enhancement blocks CAV1 expression and impairs TGFβ/TGFβR complex degradation; miR‐214‐3p promotes the non‐canonical GSK‐3β/β‐catenin axis of TGFβ signaling by targeting COX‐2 and GSK‐3β and enhancing fibroblast‐to‐myofibroblast transition; and miR‐199a‐3p mediates TGFβ‐induced inhibition of FGF7 and HGF, which are the two fibroblast‐derived growth factors that promote tissue repair. Overall, lncDNM3OS aggravates pulmonary fibrosis by promoting fibroblast differentiation into myofibroblasts by giving rise to three pro‐fibrotic miRNAs depending on cross‐talk between the TGFβ and Wnt pathways. The preclinical evidence obtained using both mouse fibrotic models, patient‐derived primary lung fibroblasts and IPF lung biopsy samples shows that lncDNM3OS antagonism function not only blocks lung fibrogenesis, but also improves established pulmonary fibrosis; thus, this function may represent a potential TGFβ‐targeted strategy for IPF.^63^, ^64^

### 3.2 lncRNA sponges for miRNA

Many lncRNAs have been confirmed to be competing endogenous RNA (ceRNA) by binding miRNAs to exert their function on target mRNAs. Despite some questions,^65^ this regulatory mode has been reported in many diseases.^66^ Huang *et al*.^67^ examined the relationship between 33,829 human lncRNAs registered in the NONCODE database and six dysregulated miRNAs in IPF, including miR‐21, miR‐29, miR‐199, miR‐31, miR‐101 and let‐7d, aiming to investigate the ceRNA mechanism in lung fibrosis. Among these, 34 lncRNAs have potential binding sites to the miRNAs as indicated by motif searching and manual comparison; however, only four lncRNAs, namely n341773, n364035, n373263 and NR‐045756, have been verified to have functional binding sites with these miRNAs. Wang *et al*.^68^ also identified a lncRNA‐miRNA‐mRNA network in IPF lung tissues according to the interactions between DElncRNA, miRNA and DEmRNA, which improves the understanding of ceRNA in IPF pathogenesis.

lncRNAs MRAK088388 and MRAK081523 are involved in the ceRNA network in bleomycin‐induced lung fibrosis in the rat, where the expression of both lncRNAs, along with their associated genes N4bp2 and Plxna4, reduces target miRNAs (miR‐29b‐3p and let‐7i‐5p).^37^ lncPCF is another lncRNA that promotes pulmonary fibrosis through a ceRNA network that involves map 3 k11, which is a key molecule controlling the cell cycle.^69^ lncPCF regulates map 3 k11 to enhance the cell cycle of activated epithelial cells by targeting miR‐344a‐5p.^70^ Three highly expressed lncRNAs (PFAR, PFAL and PFRL) were found to promote lung fibroblast proliferation, migration and differentiation in a bleomycin‐treated mice lung fibrosis model and primary mouse lung fibroblasts.^71^, ^72^, ^73^ Mechanistically, the three lncRNAs PFAR, PFAL and PFRL function as ceRNA for miR‐15a, miR‐18a and miR‐26a, respectively. The profibrotic effect of PFAR enhances the YAP1‐Twist axis by acting as an endogenous sponge of miR‐15a, leading to induction of fibrogenesis. PFAL promotes fibroblast–myofibroblast transition and extracellular matrix deposition through connective tissue growth factor by competitively binding miR‐18a. The miR‐26a‐Smad2 feedback loop mediates PFRL to induce fibroblast activation and collagen deposition. These lncRNAs lead to fibrogenesis in epithelial cells or lung fibroblasts, which suggests that knockdown lncRNA or miRNA may provide a novel strategy for intervening in lung fibrogenesis.

lncRNA H19 is the first lncRNA identified as a factor in liver development and as being under the genetic control of two *trans*‐acting loci genes, named raf and Rif, although its targets are unknown. The hypoxia‐induced H19/YB‐1 cascade modulates cardiac remodeling after infarction and may be a prognostic marker for cancer.^74^, ^75^ Several studies have shown that H19 is highly upregulated in pulmonary fibrosis *in vivo* and *in vitro*. H19 is upregulated via diminishing miR‐140 expression in fibrotic tissues from IPF patients and bleomycin‐induced pulmonary fibrosis in mice and TGFβ1‐induced HBE and A549 cells, in which the TGFβ/Smad3 signal pathway is involved in myofibroblast generation and extracellular matrix deposition. Knockdown H19 can block TGFβ/Smad3 activation, which is recognized as a “master switch” of the occurrence of fibrosis.^76^ Similarly, H19 is a direct target of COL1A1 expression by sponging miR196a or miR‐29b in TGFβ‐induced fibroblast proliferation and bleomycin‐induced lung fibrosis. H19 downregulation alleviates fibroblast activation and lung fibrosis.^77^, ^78^ Wan *et al*.^79^ further confirmed that H19 deficiency ameliorates bleomycin‐induced pulmonary infammation and fibrosis by using H19 knockout (H19−/−) mice generated by CRISPR/Cas9. Other lncRNAs, such as CHRF,^80^ ATB^81^ and ZEB1‐AS1,^82^ can regulate pulmonary fibrogenesis through sponging miRNAs.

Beside the above ceRNA network in alveolar epithelium and fibroblasts, Wang *et al*.^68^ recently discovered a ceRNA network and immune infiltration in IPF. They first identified the ceRNA network in lung tissues from 141 IPF patients. Then, by applying CIBERSORT, a newly developed bioinformatics method for the enumeration of immune cells, they found that IPF tissue samples have a higher proportion of plasma cells, M2 macrophages and resting mast cells, as well as a lower proportion of resting NK cells, monocytes and neutrophils, compared to control tissue samples. However, they did not confirm any direct correlation between immune cells and the ceRNA network in IPF tissue samples. IPF has been considered as an inflammatory disease of immune cells, although the anti‐inflammatory drug has not improved IPF patients.^83^ Perhaps many unsolved mysteries for the immune response in pulmonary fibrosis, such as cross‐talk of immune cell with other type cells, as well as alteration of the microenvironment, remain to be fully elucidated.

Additional detailed information regarding the above‐mentioned lncRNAs that regulate pulmonary fibrosis through miRNAs is summarized in Table 2.

**TABLE 2:** lncRNAs regulate pulmonary fibrosis through miRNAs

lncRNA	Regulation	Model	Targeted miRNA	Signal pathway	Cell type	Reference
MRAK088388	Up	Bleomycin‐induced fibrosis rat	miR‐29b‐3p	N4bp2		Cong *et al*.^37^
MRAK081523	Up	Bleomycin‐induced fibrosis rat	Let‐7i‐5p	Plxna4		Song *et al*.^37^
NONMMUT039556 (lnc556) and NONMMUT039865 (lnc865)	Up	Bleomycin‐induced fibrosis mouse and mouse lung fibroblast cell line L929	miR‐29b‐2‐5p	Network of lncRNA‐ circRNA‐miRNA‐mRNA	Fibroblast	Li *et al*.^42^
DNM3OS	Up	Bleomycin‐induced fibrosis mouse and TGF‐β‐treated fibroblast	miR‐199a‐5p, miR‐199a‐3p and miR‐214‐3p	TGF‐β, Wnt	Fibroblast	Savary *et al*.^63^, Jiang *et al*.^64^
n341773 n364035	Down	Lung tissues from 28 IPF patients, human fibroblasts LL29	miR‐199	Proliferation	Fibroblast	Huang et al.^67^
NR_045756	Up	Lung tissues from 28 IPF patients, human fibroblasts LL29	miR‐31		Fibroblast	Huang *et al*.^67^
n373263	Up	Lung tissues from 28 IPF patients, human fibroblasts LL29	Let‐7d		Fibroblast	Huang *et al*.^67^
PCF	Up	Bleomycin‐ induced fibrosis rat and TGF‐β‐treated RLE‐6TN cell	miR‐344a‐5p	Map 3 k11‐mediated cell cycle	Epithelial cell	Liu *et al*.^70^
PFAR	Up	Bleomycin‐induced fibrosis mouse and primary mouse lung fibroblast	miR‐15a	YAP1‐twist Axis	Fibroblast	Zhao *et al*.^71^
PFAL	Up	Bleomycin‐ induced fibrosis mouse and primary neonatal mouse lung fibroblast	miR‐18a	CTGF	Fibroblast	Li *et al*.^72^
PFRL	Up	Bleomycin‐ induced fibrosis mouse and primary neonatal mouse lung fibroblast	miR‐26a	TGF‐β/Smad2	Fibroblast	Jiang *et al*.^73^
H19	Up	Bleomycin‐ induced fibrosis mouse, lung tissues from 15 IPF patients, human type II alveolar epithelial cells A549 and human bronchial epithelium (HBE) cells	miR‐140	TGF‐β/Smad3	Epithelial cell	Wang *et al*.^76^
H19	Up		miR‐196a	COL1A1		Lu *et al*.^77^
H19	Up	Bleomycin‐ induced fibrosis mouse	miR‐29b	COL1A1 and Acta2	Epithelial cell	Tang *et al*.^78^
CHRF	Up	Silica‐induced fibrosis mouse	miR‐489	MyD88‐Smad3		Wu *et al*.^80^
ATB	Up	Silica‐induced fibrosis mouse	miR‐200c	Zeb1‐EMT	Epithelial cell	Liu *et al*.^81^
ZEB1‐AS1	Up	Bleomycin‐ induced fibrosis rat and RLE‐6TN cells	miR‐141‐3p	Zeb1‐EMT	Epithelial cell	Qian *et al*.^82^
MALAT1	Up	Silica‐induced fibrosis mouse, human bronchial epithelial cells HBE and human lung adenocarcinoma A549 cells	miR‐503	PI3K‐Akt–mTOR‐snail	Epithelial cell	Yan *et al*.^84^

### 3.3 lncRNA targets circRNA

CircRNA is a closed‐loop structure generated by pre‐mRNA back splicing. Mechanistically, circRNA can function as a ceRNA via complementary base paring similar to lncRNA. Li *et al*.^85^ first identified 67 differentially expressed circRNAs in the plasma of IPF patients using a circRNA microarray and confirmed that two upregulated circRNAs, 100,906 and 102,348, act as sponges of miRNA‐324‐5p and miRNA‐630, respectively, to control pulmonary fibrosis. Similarly, circRNA can also regulate its nearby genes such as lncRNA. CircHECTD1 controls fibroblast migration and proliferation by regulating its host gene HECTD1 in silicosis, which mediates ZC3H12A ubiquitination.^86^, ^87^, ^88^ HECTD1 encodes a protein homologous to the E6‐APC‐terminal domain‐containing E3 ubiquitinligase and mediates macrophage activation via ubiquitination of the deubiquitinating enzyme ZC3H12A.^89^ Interestingly, circZC3H4, which is transcribed from ZC3H4, plays a similar role to circHECTD1 in silicosis, possibly because ZC3H4 and ZC3H12A belong to the family of CCCH‐typezinc finger proteins.^87^

Whether lncRNA can target circRNA is still unclear. Recently, Kleaveland *et al*.^90^ found that lncCyrano, circCdr1as and two miRNAs (miRNA‐7 and miRNA‐671) form a sophisticated posttranscriptional network to exert their regulatory function in the mammalian brain. The lncCyrano directly triggers the mature miR‐7 destruction, which blocks the repression of miR‐7‐targeted mRNAs. Meanwhile, lncCyrano also enhances circCdr1as accumulation by promoting the miR‐671‐directed slicing of circCdr1as in neuron cytoplasm. In pulmonary fibrosis, cross‐talk of lncRNA with circRNA was demonstrated using whole‐transcriptome RNA sequencing to screen differentially expressed RNAs, including 585 mRNAs, 236 miRNAs, 272 lncRNAs and 74 circRNAs in lung tissues from bleomycin‐treated mice.^42^ The results of RNA‐fluorescence *in situ* hybridization, co‐localization analysis, RNA immunoprecipitation and RNA pull‐down show that lnc865 and lnc556 cross‐talk with circ949 and circ057 by targeting miR‐29b‐2‐5p, which affects phosphorylation of the target gene STAT3. The transcription factor p‐STAT3 transports into the nucleus from the cytoplasm to activate such ncRNA transcription, leading to myofibroblast proliferation and migration. These findings indicate that lncRNA can collaborate with circRNA via miRNA to establish an intricately detailed network for regulating pulmonary fibrosis. Therefore, interference of a single ncRNA may only have a limited effect. An artificially interfering lncRNA targeted multiple miRNAs has been designed to exert antitumor efficacy in hepatocellular carcinoma.^91^ Thus, targeting multiple ncRNAs is a probable direction for future pulmonary fibrosis treatment.

## 4 LNCRNA REGULATES PULMONARY FIBROSIS BY TARGETING NOT ONLY PROTEIN‐CODING GENES, BUT ALSO NON‐CODING GENES

The regulatory mechanism of lncRNA on pulmonary fibrosis is extraordinarily complicated. Several lncRNAs have been reported to contribute to pulmonary fibrogenesis by targeting not only the protein‐coding gene, but also the non‐coding gene. The anti‐fibrotic lncRNA FENDRR blocks pulmonary fibrosis and improves lung function in fibrotic human and mouse lungs and primary lung fibroblasts isolated from bleomycin‐treated mice. Mechanistically, lncFENDRR represses fibroblast activation by binding iron‐responsive element‐binding protein 1 to control iron levels and by competing with the pro‐fibrotic miR‐214.^92^ From the microenvironment, Senavirathna *et al*.^93^ have revealed that hypoxia and TGFβ1 treatment causes significant changes in 669 lncRNAs compared to 150 lncRNAs in a TGFβ1 alone group and 222 lncRNAs in a hypoxia alone group using an RNA sequencing method. Among these lncRNAs, lncFENDRR is downregulated by hypoxia and TGFβ1 in human pulmonary fibroblasts.^93^ lncMalat1can also sponge miR‐503 to control EMT through the PI3K signal pathway in silica‐induced pulmonary fibrosis.^84^ Further detailed regulatory mechanisms of lncRNAs are likely to be uncovered with the development of new sensitive profiling methods. For example, a technology named surface‐enhanced Raman spectroscopy is used to evaluate the expressed pattern of ncRNA in normal and diseased states.^94^

## 5 POTENTIAL CLINICAL APPLICATIONS

### 5.1 lncRNA as diagnostic markers

No diagnostic gold criteria or methods for IPF are available at present. The recommended methods, such as high‐resolution computed tomography, broncho alveolar lavage cellular analysis, lung biopsy or even multidisciplinary considerations, have their own limitations in clinical practice. Accordingly, a non‐invasive and specialized detection method is urgently required for clinically‐assisted diagnosis. lncRNAs have potential biomarkers in lung fibrosis because they can be easily detected in body fluids, such as blood, cerebrospinal fluid and urine, and secretory particles, including exosomes, microvesicles and apoptotic bodies.^95^ Furthermore, they are highly tissue‐specific and stable.^15^, ^16^ Although information on detecting lncRNAs in IPF is limited at present, lncITPF is detected in the peripheral blood collected from IPF patients; its expression is inversely correlated with forced vital capacity, and the area under the receiver operating characteristic curve is 0.804; therefore, lncITPF could serve as an IPF biomarker. ^39^ Other lncRNAs, such as TERRA,^25^ CDKN2B‐AS1,^47^ NR_045756^67^ and H19,^76^ can be detected in the blood of patients with IPF. However, the number of participants is insufficiently large to enable an accurate evaluation in these studies. Future work needs to expand the analysis to a larger cohort of IPF patients and determine whether the expression of these lncRNAs correlates with a worse/better patient outcome.

Fluorescence probe bioimaging technology has been utilized for an accurate, non‐invasive diagnosis in medical imaging.^96^ lncRNAs are preferred potential targets compared to traditional protein probes not only because they are smaller molecules, but also because they are more diverse than proteins. These features mean that the designed fluorescent probe is easily synthesized and can effectively penetrate cells, which could be useful for live imaging in pulmonary fibrosis. Recently, a series of bright and stable fluorescent RNAs named Peppers has been developed, and these can be inserted into different non‐coding RNA and coding RNA sequences, enabling fluorescent labeling and real‐time imaging of various RNA in living cells without affecting important functions of the target RNA, such as transcription, localization, translation and degradation.^97^ These fluorescent RNAs could be useful tools for live imaging in a variety of conditions, including pulmonary fibrosis.

### 5.2 lncRNA‐based therapy

lncRNAs are generally expressed at lower levels than protein‐coding gene.^98^ Thus, lncRNA‐based therapy has advantages compared to directly blocking mRNA or protein, which may produce adverse effects. For example, strategies to inhibit TGFβ, which is a key pro‐fibrotic regulator of fibrotic development, have had limited success because of the central nature of TGFβ in various biological processes.^99^, ^100^ Therefore, lncRNA‐based therapy could be an alternative strategy for partially inhibiting mRNA expression. On the basis of this idea, a gapmer aimed at inhibiting lncDNM3OS, which is a fibroblast‐specific downstream effector of TGFβ, has been designed to block lung myofibroblast activation.^63^, ^64^ Other drugs targeting lncRNAs, such as astaxanthin, angelica sinensis polysaccharide, astragaloside IV and astilbin, are under investigation at present. ^101^, ^102^, ^103^, ^104^ Astaxanthin alleviates lung fibrosis by blocking Smad3 nuclear translocation to inhibit lncITPF transcription.^101^ lncDANCR mediates angelica sinensis polysaccharide‐induced suppression of IPF via upregulation of FOXO3 protein levels in an AU‐binding factor 1‐dependent manner.^102^ Astragaloside IV treatment increases lncsirt1 AS and lncsirt1 AS and is validated to enhance the stability of sirt1 and increase sirt1 expression, thereby preventing IPF. ^103^

**FIGURE 1:**
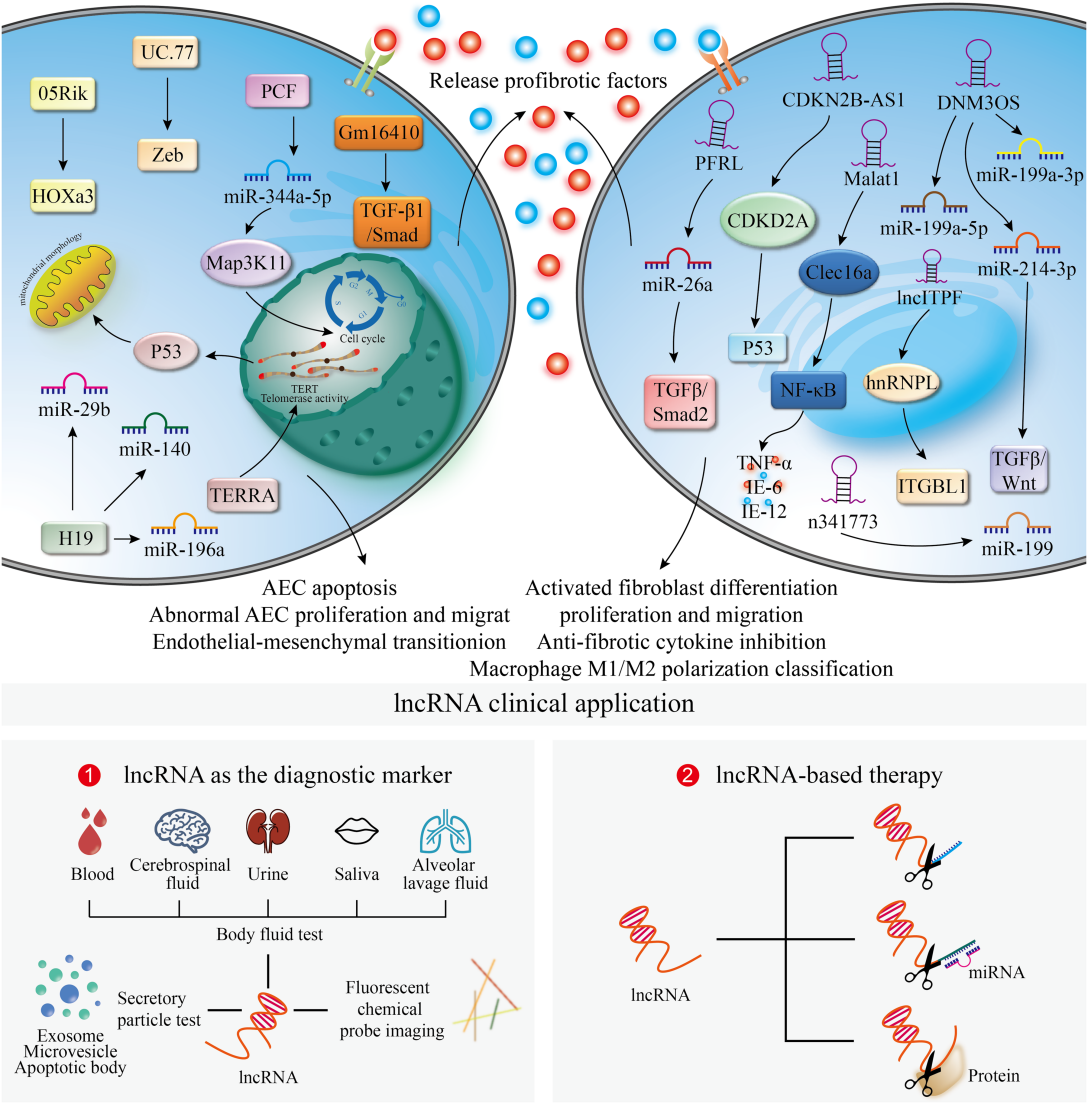
Molecular mechanisms of lncRNAs and lncRNA‐based clinical applications in pulmonary fibrosis. Aging, environmental exposure or genetic predisposition to alveolar epithelial cell results in the release of pro‐fibrotic factors, such as growth factors, cytokines, chemokines and matrix metalloproteinases, which promote abnormal and persistent fibroblast activation and remodeling, macrophage M1/M2 polarization classification, and endothelial‐mesenchymal transition. Meanwhile, many abnormal organelles, including telomere attrition, endoplasmic reticulum stress and mitochondrial dysfunction, are also involved in fibrogenesis. lncRNAs participate in these abnormal biological processes in the nucleus or cytoplasm via diverse mechanisms and thus regulate numerous signaling pathways in different cell types to control pulmonary fibrogenesis. (1) The images present the various methodologies for lncRNA as the diagnostic marker. (2) lncRNA‐based therapeutic strategies are proposed depending on their different regulatory patterns

Certainly, lncRNA‐based clinical application still has some challenges. In some cases, several lncRNAs cover the intronic and exonic sequences of their host genes, which suggests the possibility of lncRNA‐based pharmacological approaches producing off‐target effects.^105^ In addition, a few lncRNAs actually encode small proteins.^17^, ^106^ Individual lncRNA can also act as both a protein‐coding gene and an ncRNA. ^107^ Nevertheless, the protein‐coding potential of lncRNA in pulmonary fibrosis is not still documented. Despite potential issues, the spatial‐, temporal‐ and tissue‐specific expression patterns of lncRNAs suggest that this is a promising area for new targets in pulmonary fibrosis. Potential lncRNA‐based clinical applications are listed in Figure 1.

## 6 CONCLUSIONS

**FIGURE 2:**
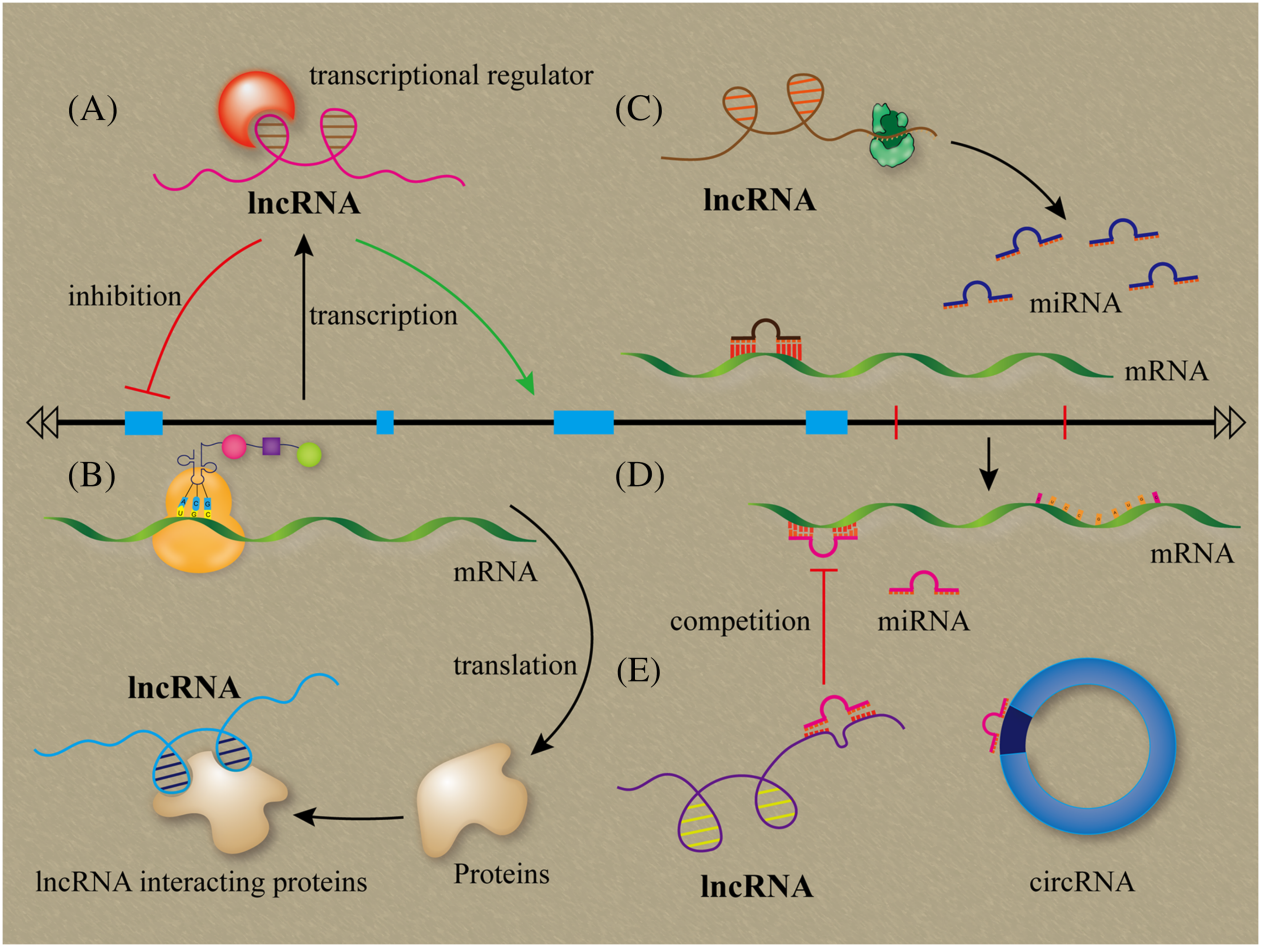
Regulatory paradigms of lncRNAs in pulmonary fibrosis. (A) lncRNA transcribed from the host gene, which affects the nearby/host genes. (B) lncRNA forms a RNA–protein complex by binding to specific protein to modulate the target gene activity. (C) lncRNA can be processed to yield miRNA or (D) function as a competing miRNA binding. (E) lncRNA cross‐talks with miRNA, circRNA and mRNA

Overall, lncRNA regulation depends on the localization and interaction of cellular compartments. As described in this review, the known mechanisms of lncRNAs in pulmonary fibrosis are attributed to five patterns: (i) lncRNA affects the nearby/host genes; (ii) lncRNA forms a RNA–protein complex to modulate target gene activity; (iii‐iv) lncRNA yields miRNA or functions as a competing miRNA with respect to binding; and (v) lncRNA cross‐talks with miRNA, circRNA and mRNA (Figure 2). Understanding these mechanisms will contribute to the development of lncRNA‐based clinical applications for pulmonary fibrosis.

## CONFLICT OF INTEREST

The authors declare that they have no conflicts of interest.

## AUTHOR CONTRIBUTIONS

Xiaodong Song, Timothy S. Blackwell and Songzi Zhang conceived, designed and reviewed the manuscript. Changjun Lv, Hongbin Chen and Dayong Yue executed the literature search and analyzed the data.

## Data Availability

The datasets used and analysed during the current study are available from the corresponding author upon reasonable request.
